# Validation of an LC-MS/MS Method for Urinary Lactulose and Mannitol Quantification: Results in Patients with Irritable Bowel Syndrome

**DOI:** 10.1155/2016/5340386

**Published:** 2016-12-14

**Authors:** Jacopo Gervasoni, Arcangelo Schiattarella, Valentina Giorgio, Aniello Primiano, Consuelo Russo, Valentina Tesori, Franco Scaldaferri, Andrea Urbani, Cecilia Zuppi, Silvia Persichilli

**Affiliations:** ^1^UOC Laboratorio Analisi I, Fondazione Policlinico Universitario A. Gemelli, Rome, Italy; ^2^Pediatria, Fondazione Policlinico Universitario A. Gemelli, Rome, Italy; ^3^UOC di Medicina Interna Gastroenterologia e Malattie del Fegato, Fondazione Policlinico Universitario A. Gemelli, Rome, Italy

## Abstract

*Aim*. Lactulose/mannitol ratio is used to assess intestinal barrier function. Aim of this work was to develop a robust and rapid method for the analysis of lactulose and mannitol in urine by liquid chromatography coupled to tandem mass spectrometry. Lactulose/mannitol ratio has been measured in pediatric patients suffering from irritable bowel syndrome.* Methods*. Calibration curves and raffinose, used as internal standard, were prepared in water : acetonitrile 20 : 80. Fifty *μ*L of urine sample was added to 450 *μ*L of internal standard solution. The chromatographic separation was performed using a Luna NH_2_ column operating at a flow rate of 200 *μ*L/min and eluted with a linear gradient from 20% to 80% water in acetonitrile. Total run time is 9 minutes. The mass spectrometry operates in electrospray negative mode. Method was fully validated according to European Medicine Agency guidelines.* Results and Conclusions*. Linearity ranged from 10 to 1000 mg/L for mannitol and 2.5 to 1000 mg/L for lactulose. Imprecision in intra- and interassay was lower than 15% for both analytes. Accuracy was higher than 85%. Lactulose/mannitol ratio in pediatric patients is significantly higher than that measured in controls. The presented method, rapid and sensitive, is suitable in a clinical laboratory.

## 1. Introduction

The gut microbiota play an important role in metabolic and immunological functions, and the impairment of its composition might alter homeostasis and lead to the development of microbiota-related diseases [1]. Irritable bowel syndrome (IBS) is a functional gastrointestinal disorder with a prevalence ranging between 10 and 30 percent [2, 3]. The principal symptoms are chronic abdominal pain associated with diarrhea and constipation.

Although this disorder is not associated with mortality, targeted treatment and therapy is desirable, especially because the causes of IBS are still partly misunderstood. There is evidence that patients with IBS have altered intestinal permeability [4, 5]; thus the evaluation of intestinal permeability together with Rome criteria and fecal calprotectin may help the discrimination between organic disease and IBS [6].

Different methods for measuring intestinal permeability are proposed.

Ussing chamber techniques, even if highly sensitive, are too invasive to be used routinely since they require various biopsies of the intestine at different levels [7].

The intestinal permeability (IP) test is an inexpensive and accurate method for evaluating the integrity of the gastrointestinal mucosa without using invasive methods such as endoscopy or radiology [7, 8].

The use of ^51^Cr EDTA not always guarantees reliable results since the use of a single molecule can detect alterations not related to the permeability alteration [8].

A useful and noninvasive marker for the evaluation of intestinal permeability may be sought in the urinary excretion of nonmetabolized sugar [9].

The evaluation of intestinal permeability based on the quantified absorption of two sugars of different sizes gives more information and higher sensitivity than using a single sugar [10]. In physiological condition the rate of absorption is about 10% for mannitol and less than 1% for lactulose. The loss of mucosal integrity should mainly cause increase of lactulose absorption with a consequent increase of lactulose-mannitol ratio (L/M ratio) in the urine sample [11].

Different laboratory procedures have been proposed for quantification of mannitol and lactulose in urine, such as spectrophotometric [12] and enzymatic [13–15] methods, gas-chromatography [16], and high performance liquid chromatography (HPLC) [17–19].

The aim of this paper was to develop and to validate a sensitive and specific liquid chromatography-tandem mass spectrometry (LC-MS/MS) method to measure mannitol and lactulose levels in urine suitable to the clinical chemistry routine. Furthermore, the method was applied for the quantification of L/M in pediatric patients suffering from IBS compared to control subjects.

## 2. Materials and Method

### 2.1. Chemicals and Reagents

Water and acetonitrile (LC-MS grade) were purchased from Merck (Merk KGaA, Darmstadt, Germany). Formic acid (98% LC-MS grade) was purchased from Baker (Mallinckrodt Baker Italia, Milano, Italia).

Lactulose, mannitol, and chlorhexidine were purchased from BioChemica (AppliChem Inc., MO, USA).

Stock solutions of mannitol (4 g/L), lactulose (4 g/L), and raffinose (1 g/L) were prepared in water and stored at 80°C.

Working solutions were prepared in water/acetonitrile (20/80, volume/volume) at concentrations of 1600 mg/L for mannitol and 800 mg/L for lactulose. Serial dilutions from working solutions were used to prepare six-point calibration curves for both mannitol and lactulose (0-50-100-200-400-800 mg/L; 0-25-50-100-200-400 mg/L, resp.) and kept at −20°C until use. Raffinose was used as internal standard (IS) at 25 mg/L in 80% acetonitrile.

### 2.2. Subject

15 patients (5–16 years, 8 males and 7 females) with a diagnosis of IBS were recruited. The diagnosis of IBS was performed using Rome III criteria [20]. Assessment of symptoms severity was performed using Visuoanalogic Scale (VAS) score. We found 8 children with IBS-D (53%), 2 with IBS-C (13%), and 5 with IBS-U (33%).

As a control group 10, apparently healthy, subjects (5–16 years, 4 males and 6 females) were recruited.

Patients with history of diabetes, thyroid disease, previous abdominal surgery, connective tissue diseases, and breath tests to lactose-positive or suffering from gastrointestinal diseases other than the IBS were excluded.

The study was approved by the institutional Ethics Committee, and an informed written consent was obtained from each subject in accordance with the principles of the Declaration of Helsinki.

### 2.3. Sample Collection and Treatment

Subjects were asked to follow a lactulose- and mannitol-free diet 24 h before the analysis in order to reduce mannitol concentration in the basal urine sample. Urine basal sample was collected after an overnight fasting; then, the patients drank a solution containing 5 gr of lactulose and 1 gr of mannitol in 120 mL of deionized water. Urine samples were collected for the next 6 h. In the collecting tube, according to the literature data, 1 mL of chlorhexidine (1 mg/mL) was added as antimicrobial agent. Total urine volume was measured, and several 1.0 mL aliquots were stored at −20°C until analysis.

Urine samples were allowed to thaw at room temperature, then stirred for 1 min using a vortex mixer, and then were centrifuged at 5,000*g* for 4 min to remove the sediment according to the laboratory procedure.

To 50 *μ*L of urine samples, controls and standards were added 450 *μ*L of IS solution and, after being mixed, a 200 *μ*L aliquot was transferred into a glass vial for the injection to HPLC-MS/MS.

### 2.4. Instrumentation

The LC-MS/MS system consisted of HPLC and autosampler Accela (Thermo Fisher, Palo Alto, CA, USA) and a triple quadrupole mass spectrometer TSQ Quantum Access (Thermo Fisher, Palo Alto, CA, USA) equipped with an electrospray ion source.

### 2.5. Chromatographic Conditions

The HPLC separation was performed using a 150 × 2 mm, Luna 5 *μ*m NH_2_ 100 Å column (Phenomenex, USA) operating at a flow rate of 300 *μ*L/min, and eluted with a 4 min linear gradient from 70 to 30% acetonitrile in water. The oven temperature was set at 40°C. The injection volume was 10 *μ*L, and the total analysis time was 9 min.

### 2.6. Mass Spectrometer Conditions

The ESI source operates in negative mode. The capillary voltage was set to 3400 V at a temperature of 310°C. The source of the gas was set as follows: sheath gas pressure, 40 (arbitrary units); auxiliary gas pressure, 5 (arbitrary units); ion sweep gas pressure, 0 (arbitrary units). Argon was used as the collision gas at a pressure of 1.5 mTorr.

Each selected reaction monitoring (SRM) transition was collected at resolution of 0.7 amu full width half maximum (FWHM) in the first quadrupole, with a scan time of 0.1 s. The tube lens and collision settings were established individually for each compound for SRM detection. The conditions (Table 1) for the detection of lactulose, mannitol, and raffinose were obtained by direct infusion of a standard solution (10 *μ*g/mL) in line with the HPLC at initial mobile phase conditions.

### 2.7. Method Validation

To validate the method the following parameters were assessed: linearity, LOQ, imprecision, accuracy, recovery, and matrix effect. Linearity and limit of quantification (LOQ) were evaluated by measuring, in triplicate, serially diluted solutions of lactulose and mannitol from the stock solution. The imprecision of the method was evaluated using a urine pool (with lactulose and mannitol not detectable, hereunder named QC0), spiked with three different amounts of sugars (50, 100, and 200 mg/L for lactulose and 100, 400, and 600 mg/L for mannitol; see Table 2). The three samples were also used as quality control samples (QC1; QC2; QC3). For the evaluation of intra-assay imprecision, each QC was prepared according to the protocol and measured eight times in the same analytical run, while the interassay imprecision was evaluated by measuring in duplicate the same QC samples for ten consecutive days. The accuracy was evaluated using ten replicates of QC1; QC2; QC3 and expressed as bias%: ([determined value/theoretical value] × 100%). For the recovery study, the urine pool (QC0) was divided into two tubes. The first aliquot (sample 1) was added with different amount of lactulose and mannitol (for the concentration values see Table 2) and treated according to the protocol. The second aliquot (sample 2) was treated according to the protocol and then enriched with the same amount of lactulose and mannitol, in order to achieve the same final concentrations in the two aliquots. The samples were then injected into the LC-MS/MS system. The recovery rate was calculated as the average of [Lactulose]_sample 1_/[Lactulose]_sample 2_ and [Mannitol]_sample 1_/[Mannitol]_sample 2_ and expressed as %.

For the evaluation of matrix effect, the QC0 was treated according to the protocol and after that enriched with different amount of lactulose and mannitol (50, 100, and 200 mg/L for lactulose and 100, 400, and 600 mg/L for mannitol). The same amount of lactulose and mannitol was added to the mobile phase in order to have the same final concentration as the enriched urine pool. The samples were then injected into LC-MS/MS system. The results were expressed as the ratio of the areas of each analyte obtained in urine and in mobile phase and expressed as %.

The stability of lactulose, mannitol, and raffinose was assessed by analyzing in duplicate five samples immediately after preparation and after 24 and 48 h stored at 4°C and −20°C. The mean concentration at each level should be within ± 15% of the nominal concentration.

Reference ranges were evaluated on 10 urinary samples from apparently healthy subject.

Data acquisition and quantitative analysis were carried out using the mass spectrometer software (Excalibur 2.0.7, Thermo Fisher, Palo Alto, CA, USA). Statistical analysis was performed using GraphPad Prism 4.00 (GraphPad Software, Inc., CA, USA) and Microsoft Excel 2007 (Microsoft Office 2007).

## 3. Results

**Figure 1:**
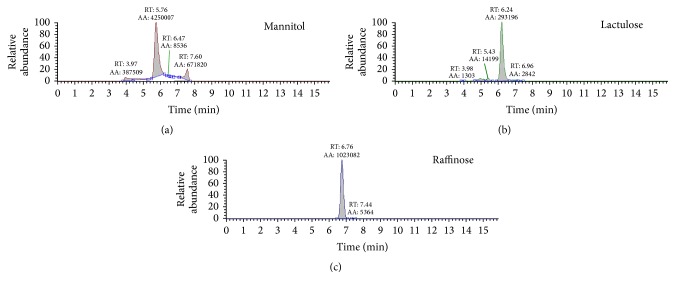
Typical SRM chromatogram for mannitol, lactulose, and IS raffinose in a urine sample.

Figure 1 shows a typical SRM chromatogram for the lactulose and mannitol and their internal standard raffinose for a urinary sample. The figure shows an adequate chromatographic separation of the three molecules and as evident no interferences were observed.

Limit of quantification was 10 mg/L for mannitol and 2.5 mg/L for lactulose. The assay was linear up to 1000 mg/L for mannitol and up to 1000 mg/L for lactulose. During method development, eight calibration curves were analyzed over a period of three weeks and *R* ^2^ was always higher than 0.99 for both analytes. Furthermore, the calibration curve parameters (slope and intercept) were compared to that obtained with urinary calibration curves. Results indicate a good overlapping of calibration points.

Precision and accuracy results are summarized in Table 2. The within-run precision and accuracy ranged from 2.9 to 5.1% and 85.6 to 96.8%, respectively. The between-run precision and accuracy ranged from 5.1 to 12.4% and 85.8 to 96.7%, respectively.

Recovery ranged from 93.5 to 118.6% for the two sugars.

The mean peak areas of lactulose and mannitol in mobile phase and in urine are not significantly different. In fact, the signal intensities of lactulose and mannitol when urinary matrix (containing chlorhexidine) was injected are completely comparable with that of the same experiment when mobile phase was injected. Matrix effect for both lactulose and mannitol was lower than 20%. Moreover this indicates that chlorhexidine, even if present in different concentration in the various urinary samples (because of the different volumes collected), did not interfere in mannitol and lactulose signals.

Mean basal lactulose and mannitol concentrations were, respectively, 4.5 ± 1.3 mg/L and 35 ± 20 mg/L without significant differences between patients and controls.

The average of the L/M ratio of the control subjects was 0.014 with a standard deviation of 0.007. The cut-off value (M + 2SD) was 0.03 and is completely comparable to data reported in the literature.

In Table 3 L/M ratios of each control and of each patient were reported.

**Figure 2:**
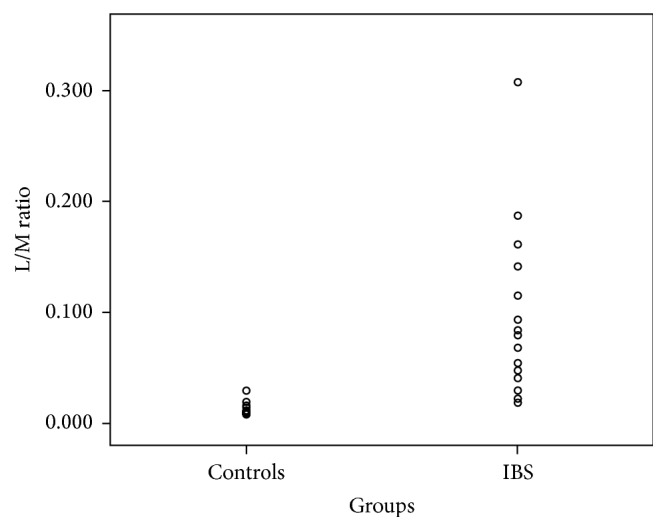
L/M ratio in controls and IBS patients (*p* < 0.05).

The average of the L/M ratio in patients with IBS was 0.096 with a standard deviation of 0.078. The statistical analysis of the differences between the L/M ratio of controls versus patients with IBS was statistically significant (*p* < 0.05) (Figure 2). Children with IBS-D had an increased intestinal permeability compared to the other IBS subgroups: IBS-D 1.19 ± 1.01 versus IBS-C and IBS-U 0.48 ± 0.40.

No gastrointestinal adverse event (vomiting, diarrhea, or nausea) was observed in any child.

## 4. Discussion

Defect of barrier function that lead to damage of the epithelial layer structure may contribute to intestinal diseases. IBS in particular is very common disease with a not clear pathophysiology. Intestinal permeability seems to play a major role in the onset and severity of related symptoms but few studies have clarified the mechanisms [21, 22].

The intestinal permeability tests represent a valid, economical, and simple tool for assessing the integrity of the intestinal barrier function and for the identification of alterations of the intestinal mucosa. Lactulose and mannitol ratio (L/M ratio) is a rapid and simple test of intestinal permeability for the assessment of intestinal barrier integrity, frequently used in clinical practice [18, 23]. Several chromatographic methods have been described for the analysis of lactulose and mannitol using different detection systems [16–19]. The HPLC coupled to mass spectrometry is the ideal solution for its high selectivity, sensitivity, and productivity.

One of the biggest problems in the mass spectrometry analysis is to minimize the matrix effect. Possible strategies to be adopted are as follows: a sample purification or a more efficient chromatographic separation [21].

In the case of urinary samples, thanks to the minimal protein concentration, it is possible to perform only a sample dilution before the injection but this procedure reduces the interference due to the matrix components only in part.

The chromatographic separation with an amine column allows adequately retaining the analytes separating them from the matrix components eluting near the void time which are mainly responsible for the matrix effect.

The amine column does not guarantee the performances of the more used C18 columns; in fact the average life observed for this application is about 400 injections, after that the CQ, repeated at each session, showed a loss of resolution for the chromatographic peak.

The choice of using a relatively long chromatographic run allows simplifying the procedure of preparation to a minimum and optimizing the work of the laboratory technician. It is possible in this way to prepare a large number of samples in a relatively short time and then perform the analysis LC-MS/MS during the night. The ESI source in negative ionization and the triple quadrupole analyzer in SRM mode ensure high specificity further reducing possible interference of other substances and also consequently increasing the sensitivity. The validation study of the method in addition to the preliminary data on real samples of urine has shown that the method fully meets the criteria required by the guidelines of the European Medicines Agency [24]. The described method allows accurately determining the urinary concentration of lactulose and mannitol. In the present study the reference intervals were evaluated on a group of pediatric subjects in order to compare them with a group of pediatric patients with IBS. The results of L/M ratio are significantly elevated in patients with IBS. The comparison between the two groups highlights the presence of an alteration of intestinal permeability in patients with IBS. In fact, the ratio of the fractions excreted lactulose of mannitol (L/M ratio) observed in urine samples of patients with IBS was significantly higher than the ratio in healthy subjects. Moreover, IBS-D children had higher degree of severity of abdominal pain (as measured by VAS score, data not shown) compared to the other subgroups suggesting higher intestinal permeability in this subgroup. As a matter of fact intestinal permeability test alone is not diagnostic of IBS and at the moment is not routinely performed, remaining confined to the research setting.

Moreover, this method allows an easy evaluation of intestinal permeability in pediatric subjects to whom it is not possible using ^51^Cr-EDTA test.

As future perspective, we would like to confirm the results of this study on a larger number of individuals to better define the cut-off values related to the various pathologies. Moreover, it would be useful to divide the study subjects in two groups (preadolescents and adolescents) to better define if the different developmental stage of the intestinal barrier may potentially affect lactulose or mannitol measurements. It is even more interesting to clarify if both aging and gut microbiota may influence intestinal permeability.

## Figures and Tables

**Table 1:** MS method parameters. The monitored precursor ion along with the corresponding product ion and collision energy (eV) and T-lens (V) are also shown.

Analyte	Precursor ion mass (*m/z*)	Production mass (*m/z*)	Transition	Collision energy (V)	T-lens (V)
Mannitol	181.0	89.1	Quantifier	21	34
	181.0	101.1	Qualifier	21	34
	181.0	119.2	Qualifier	21	34
	181.0	163.0	Qualifier	21	34

Lactulose	341.2	100.9	Qualifier	14	43
	341.2	160.9	Quantifier	14	43

Raffinose	503.1	220.7	IS	21	77

**Table 2:** Between-run and within-run accuracy and precision.

Analyte	Nominal concentration (mg/L)	Imprecision		Accuracy	
		Intra-assay CV (%)	Interassay CV (%)	Intra-assay (%)	Interassay (%)
Mannitol	100	5.1	12.1	96.4	95.9
	400	4.9	11.5	93.2	94.7
	600	3.8	12.4	96.8	96.7

Lactulose	50	2.9	5.1	85.6	85.8
	100	4.5	5.6	91.3	90.9
	200	4.2	6.3	94.3	94.3

**Table 3:** L/M ratio of each urinary sample of controls and patients.

Controls	L/M ratio	IBS	L/M ratio
1	0.010	1	0.307
2	0.010	2	0.054
3	0.009	3	0.115
4	0.029	4	0.018
5	0.014	5	0.083
6	0.019	6	0.029
7	0.008	7	0.187
8	0.016	8	0.041
9	0.009	9	0.022
10	0.011	10	0.047
		11	0.068
		12	0.093
		13	0.141
		14	0.080
		15	0.0161
